# Citrullinated fibronectin inhibits apoptosis and promotes the secretion of pro-inflammatory cytokines in fibroblast-like synoviocytes in rheumatoid arthritis

**DOI:** 10.1186/ar4112

**Published:** 2012-12-10

**Authors:** Lieying Fan, Qiang Wang, Rongqing Liu, Ming Zong, Dongyi He, Hui Zhang, Yuanyuan Ding, Jianwei Ma

**Affiliations:** 1 Department of Clinical Laboratory, Shanghai East Hospital, School of Medicine, Tong Ji University, 150 Ji Mo Road, Shanghai 200120, PR China; 2 Department of General Surgery, Shanghai Zhabei District Central Hospital, 619 Zhong Hua Xin Road, Shanghai 200072, PR China; 3 Department of Rheumatology, General Hospital, Ningxia Medical University, 804 Shengli South Road, Yinchuan 750004, PR China; 4 Department of Rheumatology, Guanghua Hospital of Integrative Medicine, 540 Xin Hua Road, Shanghai 200052, PR China

## Abstract

**Introduction:**

Rheumatoid arthritis (RA) is characterized by synovial lining hyperplasia, in which there may be an imbalance between the growth and death of fibroblast-like synoviocytes (FLSs). Antibodies against citrullinated proteins are proposed to induce RA. This study aimed to investigate the pathogenic role of citrullinated fibronectin (cFn) in RA.

**Methods:**

The distribution of fibronectin (Fn) and cFn in synovial tissues from RA and osteoarthritis (OA) patients was examined by immunohistochemical and double immunofluorescence analysis. FLSs were isolated from RA and OA patients and treated with Fn or cFn. Apoptosis was detected by flow cytometry and TUNEL assay. The expression of survivin, caspase-3, cyclin-B1, Bcl-2 and Bax was detected by real-time PCR. The secretion of proinflammatory cytokines was measured by ELISA.

**Results:**

Fn formed extracellular aggregates that were specifically citrullinated in synovial tissues of RA patients, but no Fn deposits were observed in those of OA patients. Fn induced the apoptosis of RA and OA FLSs while cFn inhibited the apoptosis of RA and OA FLSs. Fn significantly increased the expression of caspase-3 and decreased the expression of survivin and cyclin-B1 in FLSs from RA and OA patients. cFn significantly increased the expression of survivin in RA FLSs. Furthermore, cFn increased the secretion of TNF-α and IL-1 by FLSs.

**Conclusions:**

cFn plays a potential pathophysiologic role in RA by inhibiting apoptosis and increasing proinflammatory cytokine secretion of FLSs.

## Introduction

Rheumatoid arthritis (RA) is a chronic systemic autoimmune disease characterized by persistent inflammation of the synovial tissues of the joints, resulting in the loss of joint function, morbidity and premature mortality. Fibroblast-like synoviocytes (FLSs) play important role in the initiation and perpetuation of RA [1]. FLSs are characterized by the resistance to apoptosis and the consequent overexpansion and destruction of articular cartilage.

Anti-cyclic citrullinated protein (anti-CCP) antibodies belong to the family of anti-fillagrin autoantibodies [2]. Anti-CCP antibodies are produced locally in the synovium of RA patients [3]. These antibodies specifically recognize the proteins containing citrulline amino acid residues, which is generated via post-translational modification of arginine residues by peptidylarginine deiminase (PADI) [4,5]. Arginine residues often play a central role in the structural integrity of a protein, due to their ability to participate in ionic interactions with negatively charged amino acid side chains, substrates, and cofactors, and form multiple hydrogen bonds to the peptide backbone and other amino acid side chains [6]. Citrullination could destroy the ionic interactions, interfere with hydrogen bonds, and create new interactions. Therefore, the conversion of arginine into citrulline may lead to the changes in protein structure and function. Notably, the citrullinated forms of fibrinogen, fibronectin (Fn), fibrin, vimentin, collagen type II and α-enolase are common in the inflamed synovium and citrullinated fibrinogen, citrullinated fibronectin (cFn), citrullinated fibrin and citrullinated vimentin in the inflamed synovium and plasma have been considered as important citrullinated autoantigens in RA [4,7-12].

Fn comprises a large family of isomeric glycoproteins characterized by repeated amino acid units that form domains. These domains interact with various components of extracellular matrix (ECM), integrin and growth factors, which play critical roles in various physiological processes, including cell adhesion, migration, proliferation, differentiation, wound healing, fibrosis and hemostasis [13]. Fn has been shown to be synthesized locally by FLSs [14]. High level of Fn in the synovial fluid was positively correlated with the progression of joint destruction [15,16]. In addition, significant amount of cFn was present in RA synovial tissue where they formed extracellular aggregates [11].

To further elucidate the pathogenic roles of the citrullinated autoantigens, in the present study we isolated FLSs from the synovial tissues obtained from RA and osteoarthritis (OA) patients and exposed them to cFn or Fn. The results showed that cFn inhibited the apoptosis and promoted the secretion of proinflammatory cytokines in FLSs from RA patients, suggesting the pathogenic role of cFn in RA.

## Materials and methods

### Patients and controls

Synovial tissues were obtained from eight RA patients (two males, six females, median age 58 years, range 48 to 74 years) and six OA patients (three males, three females, median age 60 years, range 48 to 77 years) who underwent knee arthroscopic or replacement surgery. The tissue samples were immediately put into 1640 medium and processed within 4 h for FLSs culture and histological and immunohistochemical analysis. All patients fulfilled the American College of Rheumatology (ACR) criteria for the diagnosis of RA and OA. Informed consent was obtained from all patients and the study protocol was approved by Ethics Committee of Shanghai East Hospital.

### Isolation and culture of FLSs

Synovial tissues were minced into pieces of 2 to 3 mm in size and spread on the bottom of cell culture flasks in 1640 medium at 37°C for 6 h. Next, the tissues were incubated with complete 1640 medium supplemented with 10% fetal calf serum in a humidified atmosphere containing 5% CO_2_. The medium was changed every three to five days and non-adherent tissue pieces were carefully removed. FLSs were grown further over four to six passages. To characterize the cytological phenotype of synovial cultures, the third passage cells were stained with mouse mAb against human CD14 and CD90 (eBioscience, San Diego, CA, USA) and showed 2.8% CD14 and 97.0% CD90 expression as measured by flow cytometry.

### *In vitro *citrullination of Fn

Native human plasma Fn (Sigma-Aldrich, St Louis, MO, USA) at a final concentration of 0.5 mg/ml was incubated with 5 units/ml of PADI from rabbit skeletal muscle (Sigma-Aldrich, St Louis, MO, USA) in working buffer (100 mM Tris-HCl, 5 mM CaCl_2_, pH 7.4) at 37°C for 24 h. The reaction was stopped by the addition of 20 mM EDTA. The citrullination of Fn was detected by Western blot analysis with antibody against modified citrulline residues (anti-MC, Abcam, Cambridge, MA, USA).

### Immunohistochemical and immunofluorescence analysis

Synovial tissue samples were paraffin-embedded and cut into 5 μm sections. Sections were deparaffinized and rehydrated using standard procedures, and were stained with hematoxylin and eosin (H & E). The sections were incubated with mouse monoclonal antibody against human Fn (1:1000, Abcam, Cambridge, MA, USA) or rabbit polyclonal antibody against modified citrulline residues (anti-MC, 1:500, Abcam, Cambridge, MA, USA) at 4°C overnight. Next the sections were incubated with second antibody (EnVision™ Detection Kit, Dako, Glostrup, Denmark) for 30 min at room temperature. Immunoreactive signals were visualized using DAB. For double immunofluorescence staining, the sections were incubated with Fn antibody or anti-MC antibody at 4°C overnight, then incubated with anti-rabbit immunoglobulin G (IgG) fluorescein isothiocyanate (FITC) (1:50, eBioscience, San Diego, CA, USA). and anti-mouse IgG PE (1:100, eBioscience, San Diego, CA, USA) at 37°C for 30 min. After rinsing with PBS, the sections were observed under fluorescence microscope.

### Flow cytometry analysis of apoptosis

FLSs were stained with FITC-conjugated annexin V and propidium iodide (PI). FLSs were seeded in 24 well plates at 1 × 10^5 ^cells/well in 500 μl media and treated with 2 μg/ml Fn or cFn at 37°C for 72 h. Next, FLSs were trypsinized and collected for the detection of apoptosis by using Annexin V-FITC Apoptosis Detection Kit (eBioscience, San Diego, CA, USA). Briefly, FLSs were washed twice with cold PBS and resuspended in 500 μL binding buffer (10 mM HEPES-NaOH pH 7.4, 140 mM NaCl, 2.5 mM CaCl_2_) at a concentration of 1 × 10^6 ^cells/ml. After the addition of 5 μl Annexin V-FITC solution and PI (1 μg/ml), the cells were incubated for 15 min at room temperature and then analyzed by flow cytometer (Beckman Coulter, Fullerton, CA, USA).

### TUNEL assay

FLSs were seeded in six-well plates containing coverslips at 2 × 10^5 ^cells/well and treated with Fn or cFn (2 μg/ml) for 48 h. Apoptotic cells were detected by using In Situ Cell Death Detection Kit according to the manufacturer's instructions. Briefly, the cells were fixed with 4% paraformaldehyde for 1 h at room temperature and permeabilized with 0.1% Triton X-100. Next the cells were incubated with terminal deoxyribonucleotidyl transferase mediated dUTP nick-end labeling (TUNEL) reaction mixture for 1 hr at 37°C in the dark. For positive controls, the cells were incubated with DNase I (grade I; 3 to 3,000 U/ml in 50 mM Tris-HCl pH 7.5, 1 mg/ml BSA) for 10 min at 15 to 25°C to induce DNA strand breaks before labeling procedures. For negative controls, terminal transferase was omitted from the reaction mixture. All samples were directly analyzed under fluorescence microscope.

### ELISA

Human TNF-α, IL-1 and IL-17 levels were measured in the supernatant of FLSs using commercially available kits (R&D systems, Minneapolis, MN, USA) according to the manufacturer's instructions. The optical density (OD) of the samples was read using a microplate reader (Bio-Rad, Hercules, CA, USA) at a wavelength of 450 nm. A standard curve was generated using the OD values of standard solution for TNF-α, IL-1 and IL-17 concentration estimation.

### Quantitative RT-PCR analysis

Total RNA was extracted from FLSs using TRIzol (Invitrogen, Carlsbad, CA, USA) and reverse transcription was performed using first strand cDNA Synthesis Kit (Takara, Dalian, China) according to the manufacturer's instructions. Real-time PCR was performed using Premix Ex Taq SYBR Green PCR (Takara, Dalian, China) according to the manufacturer's instructions on an ABI PRISM 7300 (Applied Biosystems, Foster City, CA, USA). The sequences of the primers were as follows: PADI4 5'-CACAGCTCTGGTTGGCTTCA-3', 5'-CTGCACGTCCTTCAGCATCA-3'; RANKL 5'-ACCAGCATCAAAATCCCAAG-3', 5' -CCCCAAAGTATGTTGCATCC-3'; Survivin 5'-TGCCTGGCAGCCCTTTCTCA-3', 5'-TGGCACGGCGCACTTTCTTC-3'; Caspase-3 5'-TGGAACAAATGGACCTGTTGA-3', 5'-TAATAACCAGGTGCTGTGGAGT-3'; Cyclin-B1 5'-CAGTCAGACCAAAATACCTACTGGGT-3', 5'-ACACCAACCAGCTGCAGCATCTTCTT-3'; Bcl-2 5'-AGTTCGGTGGGGTCATGTGTG-3', 5'-CTTCAGAGACAGCCAGGAGAAATC-3'; Bax 5'-TTCTGACGGCAACTTCAACTG-3', 5'-TGAGGAGGCTTGAGGAGTCTC-3'; β-actin 5'-TGACTTCAACAGCGACACCCA 3', 5' -CACCCTGTTGCTGTAGCCAAA -3'. β-actin was used as internal control.

### Western blot analysis

Whole cell lysates were prepared from about 2 × 10^5 ^cells by homogenization in the lysis buffer and subsequent centrifugation at 14,000 rpm for 15 min. The protein concentration in the supernatant was determined using the Bradford method (Bio-Rad, Hercules, CA, USA). Protein samples were separated on 10% SDS-PAGE and then transferred onto nitrocellulose membranes (Amersham Pharmacia Biotech, Uppsala, Sweden). The membranes were incubated with primary antibody such as anti-MC Ab, (Abcam, Cambridge, MA, USA), anti-PADI4 (Abcam, Cambridge, MA, USA) or anti-receptor activator of nuclear factor kappa B ligand (RANKL) (Santa Cruz Biotechnology Inc., Santa Cruz, California, USA), then incubated with horseradish peroxidase-conjugated secondary antibody. All immunoreactive proteins were visualized with SuperSignals west Pico Chemiluminescent Substrate (Thermo Scientific, Rockford, IL, USA).

### Statistical analysis

The differences between means were evaluated using multiple related samples Friedman M test. Where appropriate, further pairwise comparisons were analyzed using multiple related samples q test. SPSS16.0 program package (SPSS Inc., Chicago, IL, USA) was used for all statistical analyses. A *P *value of 0.05 or less than was considered statistically significant.

## Results

### cFn is abundant in arthritic synovial tissues

**Figure 1:**
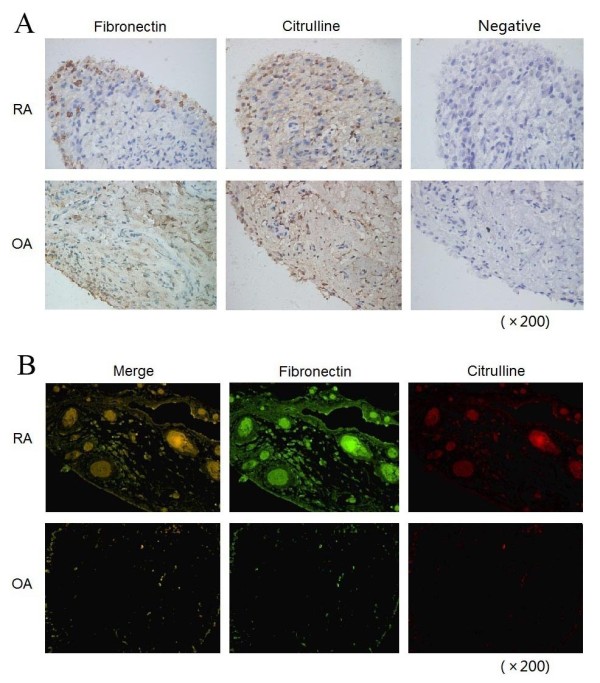
**The distribution of Fn and citrullinated proteins in arthritic synovial tissues from RA and OA patients**. **(A) **Immunohistochemistry staining. Continuous sections of RA or OA synovial tissues were probed with ant-Fn Ab and anti-MC Ab, respectively. For negative controls, primary Ab was replaced by normal serum. Original magnification: x200. **(B) **Double immunofluorescent staining. The sections of RA or OA synovial tissues were probed with anti-Fn Ab and anti-MC Ab, followed by incubation with second antibody anti-rabbit IgG FITC (green) or anti-mouse IgG PE (red). Original magnification: x200. anti-MC Ab, criteria anticitrulline (modified) antibody; FITC, fluorescein isothiocyanate; Fn, fibronectin; IgG, immunoglobulin G; OA, osteoarthritis; RA, rheumatoid arthritis.

To clarify the distribution of cFn in synovial tissues from RA and OA, we performed immunostaining and double immunofluorescent staining. Fn immunostaining was mainly detected at the outer surface of the RA synovial membrane where Fn formed a tight block but not synoviocytes (Figure 1A). Notably, the majority of such extracellular masses was also immunopositive by anti-MC staining, indicating that Fn was citrullinated. The results of double immunofluorescent staining showed similar patterns (Figure 1B). In contrast, extracellular accumulation of Fn and citrullination of proteins were undetectable in OA synovial tissue except for the deep subling region where some fibrous materials and endothelial cells were immunostained by anti-MC antibody. These results demonstrate that cFn is abundant in arthritic synovial tissues.

### Fn and cFn modulates the apoptosis of FLSs

**Figure 2:**
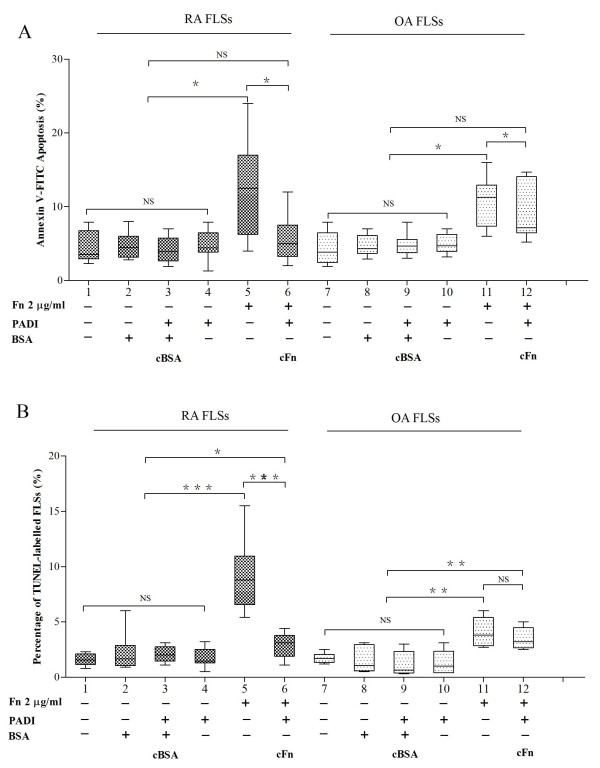
**The effects of Fn and cFn on the apoptosis of FLSs isolated from RA or OA patients**. The isolated FLSs from RA and OA patients were treated for 72 h as indicated and the apoptosis was detected by Annexin V-FITC/PI **(A) **and TUNEL analysis **(B)**. RA (*n *= 8) and OA (*n *= 6) * *P *< 0.05, ** *P *< 0.01, and NS, not significant. cFn, citrullinated fibronectin; FITC, fluorescein isothiocyanate; FLSs, fibroblast-like synoviocytes; Fn, fibronectin; OA, osteoarthritis; PI, propidium iodide; RA, rheumatoid arthritis; TUNEL, terminal deoxyribonucleotidyl transferase mediated dUTP nick-end labeling.

To evaluate the pathological significance of cFn accumulation for RA progression, we isolated FLSs from RA and OA patients as the experimental model. We prepared cFn and citrullinated BSA (cBSA) by *in vitro *citrullination using Fn and BSA as substrates, respectively. Western blot analysis with anti-MC antibody confirmed the citrullination of Fn and BSA (data not shown). Next, we treated FLSs with Fn and cFn and then detected the apoptosis of FLSs by Annexin V-FITC/PI staining and TUNEL assay. As controls we treated FLSs with BSA, cBSA or PADI and observed that BSA, cBSA or PADI had no significant effect on the apoptosis of FLSs isolated from RA or OA (Figure 2). Fn significantly increased the apoptosis of FLSs isolated from RA and OA (Annexin V-FITC/PI, *P *< 0.05; TUNEL *P *< 0.01). However, cFn attenuated the apoptosis of FLSs isolated from RA and OA (Figure 2).

**Figure 3:**
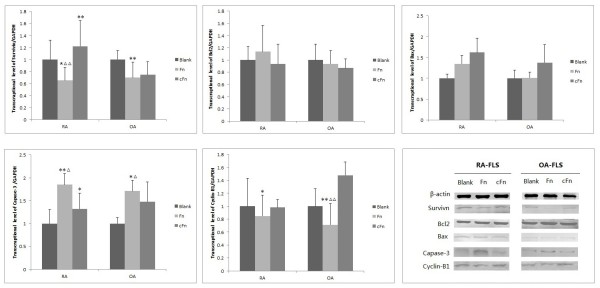
**The effects of Fn and cFn on the expression of apoptosis-related molecules in FLSs isolated from RA or OA patients**. RA or OA FLSs were treated with Fn or cFn at 2 μg/ml for 72 h and total RNA was isolated for the analysis of survivin, caspase 3, cyclin-B1, Bcl-2 and Bax mRNA levels. RA (*n *= 8) and OA (*n *= 6). * *P *< 0.05 compared with blank, ** *P *< 0.01 compared with blank. ^Δ ^*P *< 0.05 compared with cFn, ^ΔΔ ^*P *< 0.01 compared with cFn. cFn, citrullinated fibronectin; FLSs, fibroblast-like synoviocytes; Fn, fibronectin; OA, osteoarthritis; RA, rheumatoid arthritis.

To further elucidate the molecular mechanisms of Fn-induced apoptosis of FLSs, we examined the effects of Fn and cFn on the expression of apoptosis-related molecules. Fn significantly decreased the expression of survivin and cyclin-B1, but increased the expression of caspase 3 in FLSs from RA and OA (*P *< 0.05, Figure 3). In contrast, cFn significantly increased the expression of survivin in FLSs isolated from RA (*P *< 0.01). The expression of both Bcl-2 and Bax was not different in FLSs treated by 2 μg/ml Fn or cFn compared with treated control FLSs. These results suggest that survivin is implicated in Fn-induced apoptosis of FLS.

### Fn and cFn modulates the secretion of cytokines by FLSs

**Figure 4:**
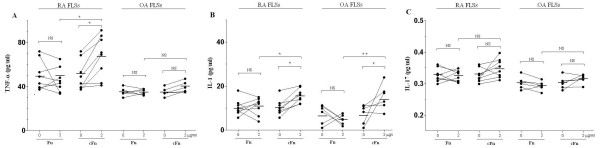
**The effects of Fn and cFn on the secretion of TNF-α, IL-1 and IL-17 by FLSs isolated from RA or OA patients**. RA or OA FLSs were treated with Fn or cFn at 2 μg/ml for 72 h and the supernatants were collected for the analysis of TNF-α, IL-1 and IL-17 levels. RA (*n *= 8) and OA (*n *= 6) * *P *< 0.05, ** *P *< 0.01, and NS, not significant. cFn, citrullinated fibronectin; FLSs, fibroblast-like synoviocytes; Fn, fibronectin; IL, interleukin; OA, osteoarthritis; RA, rheumatoid arthritis; TNF-α, tumor necrosis factor alpha.

We next investigated whether Fn and cFn have effects on the secretion of cytokines by FLSs isolated from RA or OA patients. ELISA assay showed that cFn increased the secretion of TNF-α and IL-1 by RA FLSs while Fn had no obvious effects on the secretion of TNF-α and IL-1 by RA FLSs. For OA FLSs, TNF-α secretion did not show any difference after Fn or cFn treatment (Figure 4). In addition, the secretion of IL-17 by RA and OA FLSs did not show any difference after Fn or cFn treatment (Figure 4). As the controls, we observed that BSA, cBSA, PADI had no significant effects on the secretion of TNF-α, IL-1 and IL-17 by FLSs (data not shown). Collectively, these results suggest that Fn and cFn exhibit specific effects on the secretion of TNF-α and IL-1 by FLSs isolated from RA patients.

### Fn and cFn have no effects on the expression of PADI4 and RANKL in FLSs

**Figure 5:**
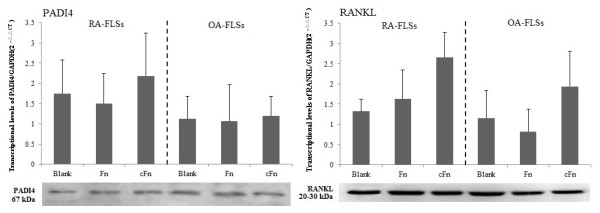
**The effects of Fn and cFn on the expression of PADI4 and RANKL in FLSs isolated from RA or OA patients**. RA or OA FLSs were treated with Fn or cFn at 2 μg/ml for 72 h, the relative mRNA levels of PADI4 and RANKL were detected by RT-PCR (upper panels), and the protein level of PADI4 and RANKL were detected by Western blot analysis (lower panels). RA (*n *= 8) and OA (*n *= 6). cFn, citrullinated fibronectin; FLSs, fibroblast-like synoviocytes; Fn, fibronectin; OA, osteoarthritis; PADI4, peptidylarginine deiminase type 4; RA, rheumatoid arthritis; RANKL, receptor activator of nuclear factor kappa B ligand; RT-PCR, reverse transcriptase polymerase chain reaction.

We assessed the expression of PADI4 and RANKL in FLSs and to further investigate the effects of Fn or cFn on the mRNA expression and protein levels of the PADI4 and RANKL in RA and OA FLSs. RT-PCR and Western blot analysis showed that Fn and cFn had no significant effects on the expression of PADI4 and RANKL at mRNA and protein levels in FLSs (Figure 5).

## Discussion

It is generally accepted that citrullinated proteins/peptides could evoke immune response leading to autoantibodies against these peptides or proteins, so called anti- cyclic citrullinated peptides (anti-CCP) [17]. The citrullinated proteins/peptides and anti-CCP are implicated in the development of RA in at least 70% patients [18]. However, current knowledge on the direct effects of citrullinated proteins on the initiation and progression in RA is extremely limited.

In this study, for the first time to our knowledge, we demonstrated that cFn inhibited the apoptosis of FLSs and augmented the secretion of proinflammatory cytokines by FLSs. These effects may explain the pathogenicity of cFn in RA. In addition, as shown in the present study, abundant Fn was mainly detected at the outer surface of the RA synovial membrane where Fn formed a tight block extracellularly. The majority of such extracellular masses were also immunopositive by anti-MC staining, indicating the abundant citrullination of Fn.

RA is a common chronic arthropathy characterized by synovial hyperplasia and progressive joint destruction. The synovial tissue is mainly composed of FLSs, which are key players in the physiopathology of RA through the local secretion of proinflammatory cytokines, inflammation mediators and proteolytic enzymes that degrade EMC and destroy the joint structure. FLSs derived from RA synovium exhibit aggressive and invasive properties. It has been shown that the proliferation rate of FLSs was similar whether they were isolated from healthy individuals, RA or OA patients [19,20]. Thus the overgrowth of FLSs population in RA is more likely due to an imbalance between cell proliferation, survival, and death. In this study we proved that cFn could inhibit the apoptosis of FLSs, which may promote their survival and participation in the pathogenic process.

Apoptosis is tightly regulated by anti- or pro-apoptotic molecules. Survivin is an important inhibitor of apoptosis that is undetectable in terminally differentiated adult tissues but overexpressed in cancer [21,22]. In the present study, we showed that Fn significantly inhibited the expression of survivin and cyclin-B1, and promoted the expression of caspase 3 in FLSs. However, cFn significantly increased the expression of survivin in FLSs isolated from RA patients. Meanwhile, the expression of anti-apoptotic molecule Bcl-2 and pro-apoptotic molecule Bax did not changed in FLSs treated by Fn or cFn. These results demonstrate that the molecular mechanisms underlying Fn/cFn-regulated apoptosis are due to survivin-dependent signal pathway. Additionally, these data are consistent with previous study reporting that the ability of Fn to promote the apoptosis of monocytes was considerably reduced after citrullination [11]. Therefore, citrullination could change the function of Fn and citrullinated autoantigens may play an important role in the promotion of rheumatoid synovial hyperplasia via the inhibition of FLSs apoptosis.

Various cytokines are known to play key roles as mediators of RA progression. In particular, TNF-α and IL-1 are located at the upstream of the cytokine cascade, and are pivotal mediators in the induction of an excessive reaction *in vivo *[23]. TNF-α appears to play important role in triggering events leading to inflammation both locally and systemically, whereas IL-1 is involved in cartilage and bone destruction [24]. In the present study we demonstrated, for the first time, that cFn promoted the secretion of TNF-α and IL-1 by FLSs from RA. This further expands the importance of the pathophysiologic role of the citrullinated autoantigens in RA.

Receptor activator of nuclear factor kappa B (RANK) and its ligand, RANKL, are members of the TNF receptor and TNF superfamilies, respectively. The RANKL/RANK system is essential for osteoclast formation [25,26]. The level of soluble RANKL was elevated in synovial fluid of RA patients and both activated T cells and FLSs express RANKL [27,28]. Page *et al. *reported that treatment of synoviocytes with TNF-α or IL-1β in combination with IL-17 is particularly potent for inducing RANKL expression [29]. In the present study, we found that treatment with cFn or native Fn did not induce significant change in RANKL expression level in RA and OA FLSs. These observations suggest that RANKL is unlikely to be responsible for the inhibiton of apoptosis and the overproduction of proinflammatory cytokines in RA FLSs treated by cFn.

PADI mediates the post-translational deimination of arginyl residues, a process named as citrullination. Several studies reported that PADI4 was extensively expressed in FLSs in the lining and sublining areas of RA synovium that was responsible for the citrullination of fibrin [30,31]. Interestingly, our recent results showed that citrullinated vimentin could promote the expression of PADI4 in FLSs [32], suggesting the possible existence of a positive feedback loop that augments the citrullination of proteins involved in RA. To investigate whether cFn could also promote PADI4 expression in FLSs, we treated FLSs with cFn and found that cFn and Fn had no significant effects on PADI4 expression in FLSs. These results suggest that the abundance of PADI4 in RA synovial tissue may be mediated by other mechanisms than cFn.

## Conclusions

Our present study demonstrated that cFn plays a potential pathophysiologic role in RA by inhibiting apoptosis and increasing proinflammatory cytokine secretion of FLSs. Insufficient apoptosis and/or enhanced proinflammatory cytokine production may contribute to the increased number of FLSs and rheumatoid synovial hyperplasia in RA joints. These findings provide new insights into the role of citrullinated autoantigens in RA. Further studies are necessary to elucidate the molecular mechanism by which citrullinated proteins contribute to the onset and progression of RA.

## Abbreviations

ACR: American College of Rheumatology; anti-CCP: anti-cyclic citrullinated protein antibodies; anti-MC Ab: criteria anticitrulline (modified) antibody; BSA: bovine serum albumin; cFn: citrullinated fibronectin; ECM: extracellular matrix; ELISA: enzyme-linked immunosorbent assay; FITC: fluorescein isothiocyanate; FLSs: fibroblast-like synoviocytes; Fn: fibronectin; IgG: immunoglobulin G; IL: interleukin; mAb: monoclonal antibody: OA: osteoarthritis; PADI4: peptidylarginine deiminase type 4; PBS: phosphate-buffered saline; PI: propidium iodide; RA: rheumatoid arthritis; RANKL: receptor activator of nuclear factor kappa B ligand; RT-PCR: reverse transcriptase polymerase chain reaction; SDS-PAGE: sodium dodecyl sulphate polyacrylamide gel electrophoresis; TNF-α: tumor necrosis factor alpha; TUNEL: terminal deoxyribonucleotidyl transferase mediated dUTP nick-end labeling.

## Competing interests

The authors have declared no conflicts of interest.

## Authors' contributions

LYF and QW designed and directed the research and drafted the manuscript. RQL and DYH collected clinical samples and data. MZ, HZ, YYD and JWM performed the experiments. All authors read and approved the final manuscript for publication.
